# Perceptions of and preparedness for cross-cultural care: a survey of final-year medical students in Ireland

**DOI:** 10.1186/s12909-024-05392-4

**Published:** 2024-04-29

**Authors:** Lesley O’Brien, Nicola Wassall, Danielle Cadoret, Aleksandra Petrović, Patrick O’Donnell, Siobhán Neville

**Affiliations:** School of Medicine, University of Limerick, Limerick, Republic of Ireland

**Keywords:** Cross-cultural care, Cultural competency, Medical education

## Abstract

**Background:**

Migration is increasing globally, and societies are becoming more diverse and multi-ethnic. Medical school curricula should prepare students to provide high-quality care to all individuals in the communities they serve. Previous research from North America and Asia has assessed the effectiveness of medical cultural competency training, and student preparedness for delivery of cross-cultural care. However, student preparedness has not been explored in the European context. The aim of this study was to investigate how prepared final-year medical students in the Republic of Ireland (ROI) feel to provide care to patients from other countries, cultures, and ethnicities. In addition, this study aims to explore students’ experiences and perceptions of cross-cultural care.

**Methods:**

Final-year medical students attending all six medical schools within the ROI were invited to participate in this study. A modified version of the Harvard Cross-Cultural Care Survey (CCCS) was used to assess their preparedness, skill, training/education, and attitudes. The data were analysed using IBM SPSS Statistics 28.0, and Fisher’s Exact Test was employed to compare differences within self-identified ethnicity groups and gender.

**Results:**

Whilst most respondents felt prepared to care for patients in general (80.5%), many felt unprepared to care for specific ethnic patient cohorts, including patients from a minority ethnic background (50.7%) and the Irish Traveller Community (46.8%). Only 20.8% of final-year students felt they had received training in cross-cultural care during their time in medical school. Most respondents agreed that they should be assessed specifically on skills in cultural competence whilst in medical school (83.2%).

**Conclusions:**

A large proportion of final-year medical students surveyed in Ireland feel inadequately prepared to care for ethnically diverse patients. Similarly, they report feeling unskilled in core areas of cross-cultural care, and a majority agree that they should be assessed on aspects of cultural competency. This study explores shortcomings in cultural competency training and confidence amongst Irish medical students. These findings have implications for future research and curricular change, with opportunities for the development of relevant educational initiatives in Irish medical schools.

**Supplementary Information:**

The online version contains supplementary material available at 10.1186/s12909-024-05392-4.

## Background

Patient-centred care is linked to improved health outcomes for patients and represents a pillar of quality in healthcare delivery [1]. However, providing patient-centred care becomes more complex with increasing diversity of patient populations. Miscommunication and misunderstandings in the clinical setting can lead to patient dissatisfaction, reduced adherence to treatment regimens and poor health outcomes [2]. Cross-cultural competence is an important factor in the ability of clinicians to deliver appropriate care to patients from different sociocultural backgrounds [1]. There have been multiple definitions of cross-cultural competence developed in the literature, and for the purpose of this study cross-cultural competence can be taken to mean a shared knowledge from collective experiences of diverse groups and the integration of behaviours and attitudes by healthcare professionals to empower them to engage effectively and collaboratively with patients from these diverse backgrounds [3]. Healthcare providers skilled in cross-cultural care can improve quality of care for minority ethnic groups and help eliminate health disparities by improving communication with patients, building trust, and overcoming gaps in understanding [2]. Therefore, it can be argued that cross-cultural competence is an essential skill for clinicians and should be included in medical school curricula.

To provide appropriate cross-cultural care, clinicians must engage in effective communication with, and provide high quality care to, patients from diverse sociocultural backgrounds [4]. Whilst there is no accepted definition of cultural competence, Betancourt et al*.* [5] described cultural competence training as specific efforts to enhance knowledge of sociocultural factors, health beliefs and behaviours held by patients, with an aim to develop skills to manage these factors in the delivery of equitable health care. Ultimately, training should help clinicians understand the impact of sociocultural factors on a patient’s health. However, there is variability in the methods, timing, and quality of this training [6]. Some institutions prioritise theory over practical skills, and many fail to address bias and disparities in healthcare [6]. Cross-cultural training requires standardisation to consistently produce culturally competent clinicians.

There has been a drive to improve cross-cultural care training in medicine, as studies in various countries have shown that both medical students and practising clinicians feel unprepared to deliver patient-centred, cross-cultural care [1, 2, 6]. Using the Cross-Cultural Care Survey (CCCS), a tool developed and validated for assessing cultural competency in medicine, Green *et. al.* [6] reported that final year Harvard medical students felt they lacked experience with diverse patient populations and experienced dismissive attitudes towards cross-cultural training from educators. As a result, they felt unprepared in many facets of delivering cross-cultural care. Medical students in Taiwan reported no improvement in preparedness to deliver cross-cultural care or address health inequities, as they progressed from preclinical to clinical training [7]. In Pakistan, researchers found that there was little difference between medical school year groups in their preparedness to care for patients with cultural customs and/or beliefs with the potential to affect clinical care [8]. In Switzerland, Casillas et al. [2] surveyed a group of healthcare providers, which included physicians and clinical nurses, and found that participants felt least prepared to care for patients whose religious beliefs affect treatment, and working in a department that provided some form of cross-cultural training was associated with higher levels of preparedness. Hudelson et al. [9] assessed the communication skills of both healthcare providers and local medical students when caring for migrant patients, which found that medical students scored lower than their qualified colleagues in clinical skills, intercultural communication skills and general intercultural skills.

There have been several studies conducted in the United Kingdom (UK) examining the cultural awareness of medical students and delivery of cultural competency training [10]. Studies show that UK medical students wish to be more aware of cultural differences in their patient population, and some students had not encountered any form of cultural competency training in their clinical curricula [11, 12]. They recommended the incorporation of cultural competency training in both clinical and didactic material [12].

In the Irish context, the health of the Irish Traveller community raises particular equity concerns. This minority ethnic group faces higher mortality rates and lower average life expectancies than the general population, likely due to factors including discrimination, and access to health and social services [13]. This group was formally recognised as an indigenous ethnic minority in 2017 [14]. Diversity in Ireland has also been increasing, which adds to the complexity of delivering patient-centred care. From 2011 to 2016, the non-White-Irish population increased at a rate three times that of the White Irish ethnic majority. Preliminary results for the 2022 census revealed a population increase of 361,671, and estimated net immigration of 190,330 [15]. More recently, over 60,000 Ukrainian refugees arrived in Ireland in less than a year, with many requiring access to health services [16]. Despite these big changes, there is no research we can find that examines the preparedness of medical students in Ireland to provide cross-cultural care*.* The Health Service Executive (HSE), Ireland’s public health and social care service, recommends that academic institutions should integrate cultural competency training into undergraduate and postgraduate medical programmes [17]. Ireland’s changing demographics necessitate effective cross-cultural training in medicine to ensure all patients receive high quality care. Varying degrees of cross-cultural training have been employed by Irish medical institutions to provide students with skills required to navigate cross-cultural consultations. It is important to gauge the effectiveness of this training, particularly as there is no national standard in this area.

The aim of this study is to examine whether final-year medical students in Ireland feel prepared to provide high-quality care to patients from diverse cultural and ethnic backgrounds. Furthermore, this study aims to explore how these students have encountered this concept in their training thus far. In addition, we sought to explore the perceptions of students regarding ethnicity and health and identify potential areas to build on in medical school curricula. Researching the student perspective can provide medical educators with information on where students are receiving training in cross-cultural care, where students are finding greatest engagement, and areas of cross-cultural care in which students feel underprepared. Teaching basic skills required for navigating cross-cultural care consultations early in a medical student’s education establishes a foundation to build upon throughout their career and aid in the delivery of equitable healthcare for all patient cohorts.

## Methods

### Population and recruitment

We recruited final-year medical students, due to graduate in 2022, from both undergraduate and graduate-entry programmes in all six medical schools within the ROI: National University of Ireland, Galway (NUIG), Royal College of Surgeons in Ireland (RCSI), Trinity College Dublin (TCD), University College Cork (UCC), University College Dublin (UCD), and University of Limerick (UL). We used social media channels to distribute the survey, posting to final year medical student groups on Facebook, WhatsApp and Instagram platforms. We included a prize draw as an incentive to participate. Ethical approval was granted by the University of Limerick Faculty of Education and Health Sciences Research Ethics Committee.

### Design and procedure

The Harvard cross-cultural care survey (CCCS) is a validated tool developed in the United States to assess cross-cultural competence in medicine and was adapted for the Irish context [6]. For example, questions were included regarding the Irish Traveller population. This cross-sectional survey design was used to collect both quantitative and qualitative data on four elements of cross-cultural care: i) preparedness, ii) skill, iii) training and education, and iv) attitudes. Information was collected on the medical training received by each participant, in addition to experiences outside of medical school. We assessed students during their final semester of medical school from January to June 2022. The survey was created using the online survey platform Qualtrics in line with the Harvard cross-cultural care survey layout. It was distributed via social media channels, and in addition, posters with a QR code link to the survey were placed in communal student settings in Irish hospitals.

Survey responses were stored and analysed using IBM SPSS Statistics 28.0. All components of the survey, including demographics, preparedness, skill, training and education, and attitudes were examined using frequency analyses. Fisher’s exact test was used to investigate statistically significant differences between gender and ethnicity groups, in reported preparedness and skill. Fisher’s Exact Test was chosen to provide accurate p values for low frequency samples in this study.

Participants were asked initially to disclose whether they were a final year medical student from the outset. Those who responded “no” were filtered out from the participation in the survey. Demographic data collected were analysed to investigate for gender and ethnicity difference in responses without cross-analysing two variables, e.g. institute and ethnicity or gender and ethnicity, to ensure confidentiality. Following completion of the survey, participants were offered the chance to participate in the prize draw. If accepted, participants were taken to a separate survey in which they were asked to include their email for a chance to win. No names were collected to protect confidentiality.

### Pilot study

A pilot study of recently qualified doctors was carried out in 2021 to further refine the CCCS for the Irish context. The survey was distributed to recent graduates from six medical schools in the ROI via social media. The pilot study was completed by 49 participants. The reliability of the survey was tested using data collected from the pilot study. Cronbach’s alpha for the subscales within the survey were (α = 0.705–0.887) indicating acceptable reliability.

## Results

A total of 105 survey responses were collected from final-year medical students across the six medical schools in the ROI. Twenty-eight responses were excluded—four were not final year medical students and twenty-four were incomplete. There was a target population of approximately 1200 students, however the true number of students viewing the survey link is not known, therefore an exact response rate cannot be calculated.

### Demographics

NUIG returned the most responses, (Table 1 (demographics), *N* = 77). There was a higher proportion of female to male participants (75.3% and 24.7%, respectively). 57 participants self-identified as “ethnic majority” (74%), 19 self-identified as “ethnic minority”, (24.7%), and one did not disclose their self-identified ethnicity (1.3%).

**Table 1:** Demographics

	Number of participants	% Total
Please indicate your medical school:		
NUIG	28	36
UL	21	27
UCD	13	17
UCC	9	12
RCSI	4	5
TCD	2	3
Prefer not to say	0	0
Please indicate the gender you identify as:		
Female	58	75
Male	19	25
Non-binary	0	0
Prefer not to say	0	0
Do you identify as an ethnic minority?		
No	57	74
Yes	19	25
Prefer not to say	1	1

Participant demographics including university, gender identity, and ethnicity identity. Total number of participants (*N* = 77)

### Preparedness

Participants were asked to evaluate their perceived level of preparedness to care for patients in the contexts presented in Table 2 (Preparedness). 80.5% of participants felt prepared to care for patients in general. 50.7% felt prepared to care for patients from ethnic minorities, and 46.8% felt prepared to care for Irish Travellers specifically. Participants felt unprepared to care for new migrant patients (62.4%), patients with limited English proficiency (57.2%), and patients whose religious beliefs may affect clinical care (57.2%). There were statistically significant differences when comparing ethnicity groups. Ethnic minority participants felt more prepared to care for patients from racial/ethnic minority backgrounds (68.42% vs 43.86%, *p* < *0.05*), patients with limited English proficiency (26.32% vs 22.81%, *p* < *0.05*), and new migrant patients (26.32% vs 17.54%, *p* < *0.05*) when compared to those who did not identify this way. There were no statistically significant differences between gender groups.

**Table 2:** Final-year medical students self-reported preparedness

Please indicate how prepared you feel to care for patients in the following:	Very unprepared	Somewhat unprepared	Neither prepared nor unprepared	Somewhat prepared	Very prepared
	N %	N %	N %	N %	N %
To care for patients in general?	2 (2.6%)	9 (11.7%)	4 (5.2%)	52 (67.5%)	10 (13.0%)
To care for patients who are members of racial and ethnic minorities?^*^	7 (9.1%)	13 (16.9%)	18 (23.4%)	34 (44.2%)	5 (6.5%)
To care for patients who are members of the Irish Traveller community?	8 (10.4%)	17 (22.1%)	16 (20.8%)	32 (41.6%)	4 (5.2%)
To care for patients who have limited proficiency in the English language?^*^	12 (15.6%)	32 (41.6%)	14 (18.2%)	16 (20.8%)	3 (3.9%)
To care for patients who are new immigrants?^*^	16 (20.8%)	32 (41.6%)	13 (16.9%)	10 (13.0%)	6 (7.8%)
To care for patients whose religious beliefs affect treatment?	14 (18.2%)	30 (39.0%)	13 (16.9%)	15 (19.5%)	5 (6.5%)
To care for patients who are LGBTQIA + ?	9 (11.7%)	15 (19.5%)	11 (14.3%)	31 (40.3%)	11 (14.3%)
To care for patients who are persons with disabilities?	6 (7.8%)	16 (20.8%)	18 (23.4%)	26 (33.8%)	11 ( 14.3%)

Participants were asked to self-evaluate their level of preparedness for each context presented above using a 5-point Likert scale from very unprepared to very prepared. % *N* = percentage frequency of number of total participants (*N* = 77)

^*^p < 0.05, representing statistically significant differences identified between self-identified ethnicity groups following Fisher’s Exact Test. No statistically significant differences were identified between gender groups

Participants were *similarly prepared* to care for other minoritised patient communities, including those who identify as LGBTQIA + (54.6%) and those with disabilities (48.1%), as compared to patients from minority ethnic backgrounds (50.7%).

### Skill

Participants were asked to evaluate their perceived level of skill in relation to the contexts presented in Table 3 (Skill). A high proportion of participants reported being skilled in adapting communication styles to fit a patient’s needs (80.5%) and building rapport with patients from ethnic backgrounds different to their own (76.6%). Participants reported a higher level of skill in identifying a patient’s understanding of spoken English (64.9%) compared to written English (42.9%). Participants reported being unskilled in working effectively with a medical interpreter (45.5%) and identifying religious beliefs and cultural customs that may affect clinical care (44.2%). Ethnic minority participants reported greater skill in identifying how well a patient understands verbal English than their ethnic majority counterparts (78.95% vs 59.65%, *p* < *0.05*). There were no statistically significant differences observed between gender groups.

**Table 3:** Final-year medical students self-reported skill

Please rate how skilled you are at each of the following:	Very unskilled	Somewhat unskilled	Neither skilled nor unskilled	Somewhat skilled	Very skilled
	N %	N %	N %	N %	N %
Identifying how well a patient can read or write English	10 (13.0%)	10 (13.0%)	24 (31.2%)	26 (33.8%)	7 (9.1%)
Identifying how well a patient understands the English that is being spoken to them^*^	4 (5.2%)	8 (10.4%)	15 (19.5%)	40 (51.9%)	10 (13.0%)
Identifying religious beliefs and cultural customs that might affect clinical care	9 (11.7%)	25 (32.5%)	20 (26.0%)	18 (23.4%)	5 (6.5%)
Adapting my communication style to accommodate a patient’s needs	4 (5.2%)	5 (6.5%)	6 (7.8%)	52 (67.5%)	10 (13.0%)
Building rapport with patients from an ethnic background different to my own	3 (3.9%)	6 (7.8%)	9 (11.7%)	44 (57.1%)	15 (19.5%)
Working effectively with a medical interpreter	17 (22.1%)	18 (23.4%)	27 (35.1%)	10 (13.0%)	5 (6.5%)

Participants were asked to self-evaluate their level of skill for each context presented above using a 5-point Likert scale from very unskilled to very skilled. % *N* = percentage frequency of number of total participants (*N* = 77)

^*^p < 0.05, representing statistically significant differences identified between self-identified ethnicity groups following Fisher’s Exact Test. No statistically significant differences were identified between gender groups

### Training and education

**Fig. 1:**
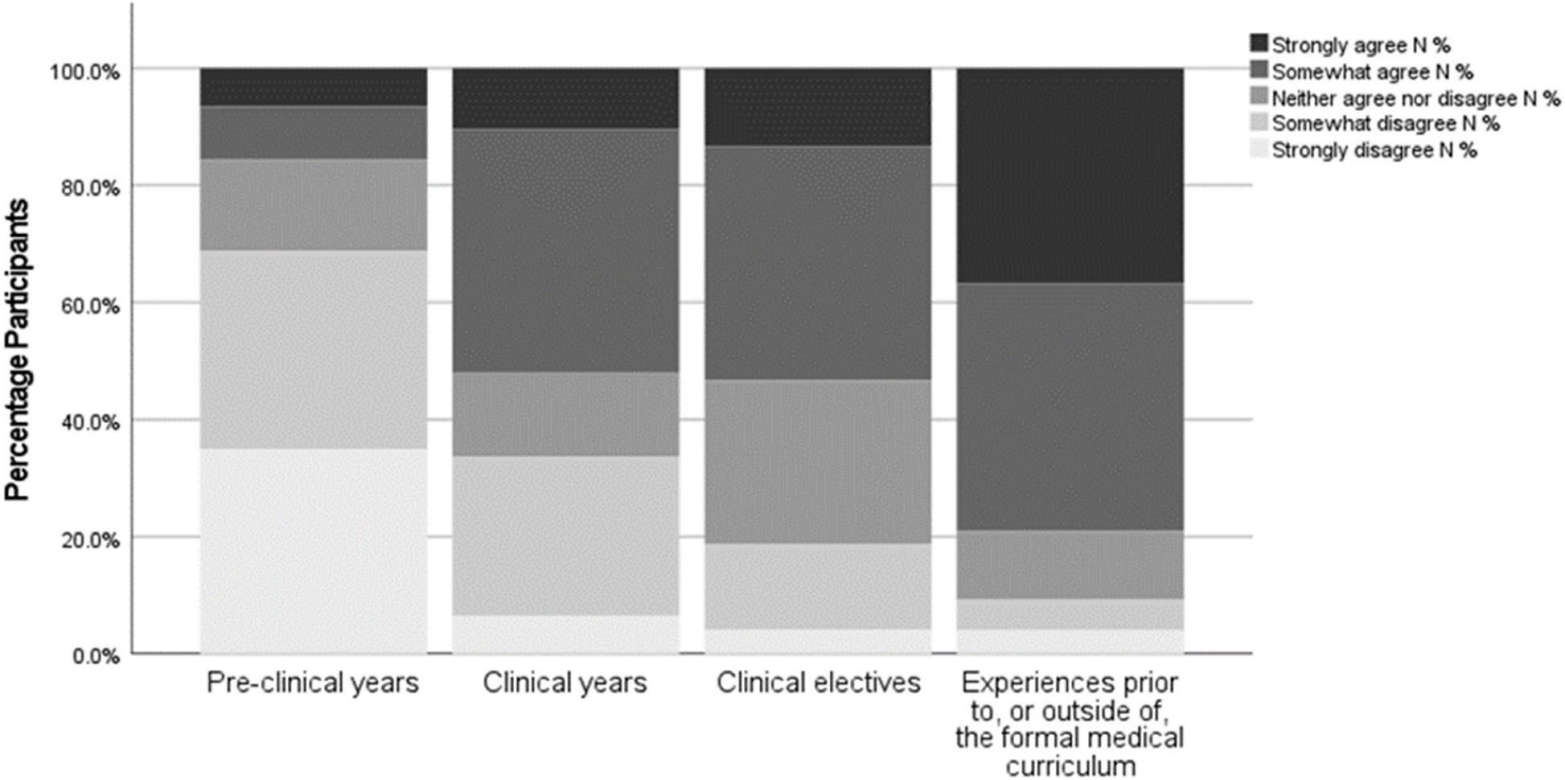
Training and Education: Experience. Participants were asked to self-evaluate the usefulness of the educational experiences presented above in preparing them to care for ethnic minority patients using a 5-point Likert scale from strongly disagree to strongly agree. % *N* = percentage frequency of number of total participants, (*N* = 77)

Participants were asked to evaluate how their educational experiences have prepared them to care for ethnic minority patients (Fig. 1). Participants identified experiences prior to, or outside of, the formal medical curriculum as the most useful in preparing them (36.8% “strongly agree”, 42.1% “somewhat agree”), followed by clinical electives (13.3% “strongly agree”, 40% “somewhat agree”) and formal clinical years (10.4% “strongly agree”, 41.6% “somewhat agree”). The pre-clinical education period (usually the first half of medical school training) was where a minority of students surveyed gained educational experience relevant to this topic, (6.5% “strongly agree”, 9.1% “somewhat agree”).

**Fig. 2:**
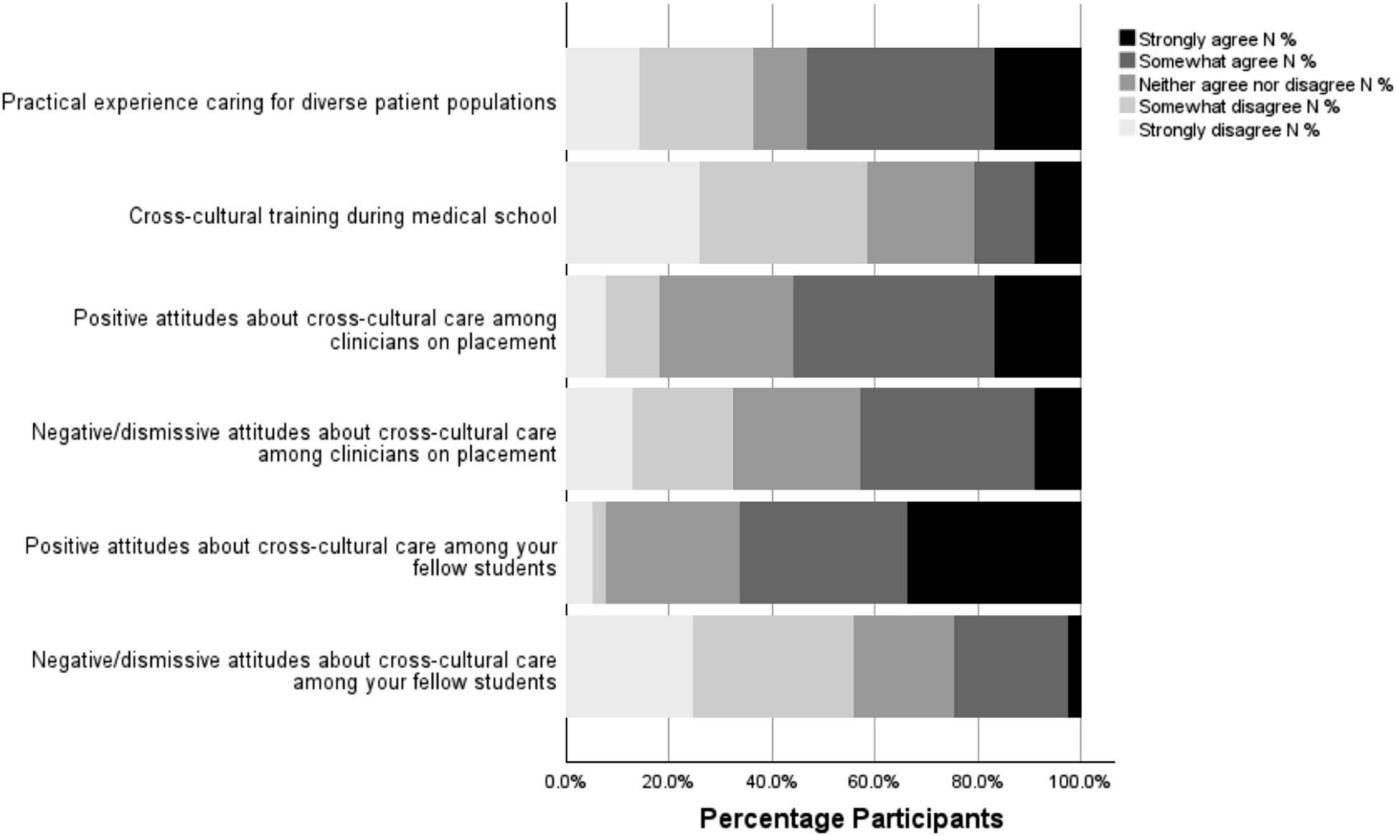
Training and Education: Exposure. Participants were asked to self-evaluate whether they had been exposed to the scenarios presented above using a 5-point Likert scale from strongly disagree to strongly agree. % *N* = percentage frequency of number of total participants, (*N* = 77)

Participants were asked to identify whether they had been exposed to various aspects of cross-cultural training whilst in their medical school (Fig. 2). Participants agreed they had practical experience caring for diverse patient populations during this time (16.9% “strongly agree”, 36.4% “somewhat agree”). A minority of participants felt they had not encountered diverse patient populations (22.1% “somewhat disagree”, 14.3% “strongly disagree”). Few agreed they had undergone cross-cultural training (9.1% “strongly agree”, 11.7% “somewhat agree”). A small majority agreed they had encountered positive attitudes to cross-cultural care amongst senior clinicians on placement, (16.9% “strongly agree”, 39% “somewhat agree”). A similar proportion encountered negative or dismissive attitudes amongst senior clinicians, (9.1% “strongly agree”, 33.8% “somewhat agree”). A majority of participants reported encountering positive attitudes towards cross-cultural care amongst their student peers (33.8% “strongly agree”, 32.5% “somewhat agree”). There were no statistically significant differences between gender or ethnic groups.

**Fig. 3:**
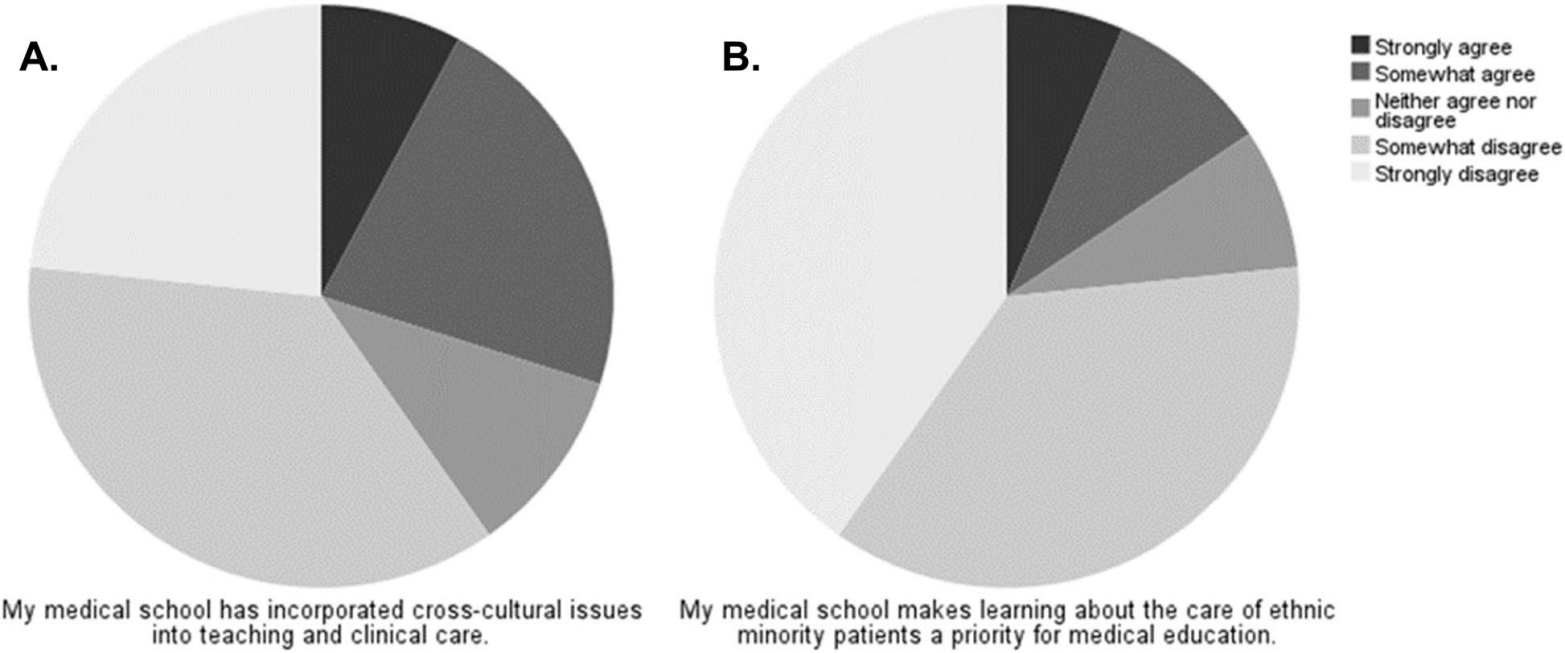
Student Perceptions on Current Cross-Cultural Training in Medical School. Participant perceptions were assessed using a 5-point Likert scale from strongly disagree to strongly agree. **A** Participants were asked whether their medical school had incorporated cross-cultural issues into teaching and clinical care. Strongly disagree (23.4%), somewhat disagree (36.4%), neither agree nor disagree (10.4%), somewhat agree (22.1%), strongly agree (7.8%). **B** Participants were asked whether they felt their medical school makes learning about the care of ethnic minority patients a priority. Strongly disagree (40.3%), somewhat disagree (36.4%), neither agree nor disagree (7.8%), somewhat agree (9.1%), strongly agree (6.5%). % *N* = percentage frequency of total participants, where *N* = 77. No statistically significant differences were identified between ethnic groups or gender groups

Participants were asked to evaluate how they felt their medical school had incorporated and prioritised the teaching of cross-cultural care. Many disagreed that their respective schools had incorporated cross-cultural issues into teaching (Fig. 3A) (36.4% “somewhat disagree”, 23.4% “strongly disagree”). Similarly, they disagreed that their medical school had made the care of ethnic minority patients a priority for medical education (Fig. 3B) (36.4% “somewhat disagree”, 40.3% “strongly disagree”).

### Attitudes

**Fig. 4:**
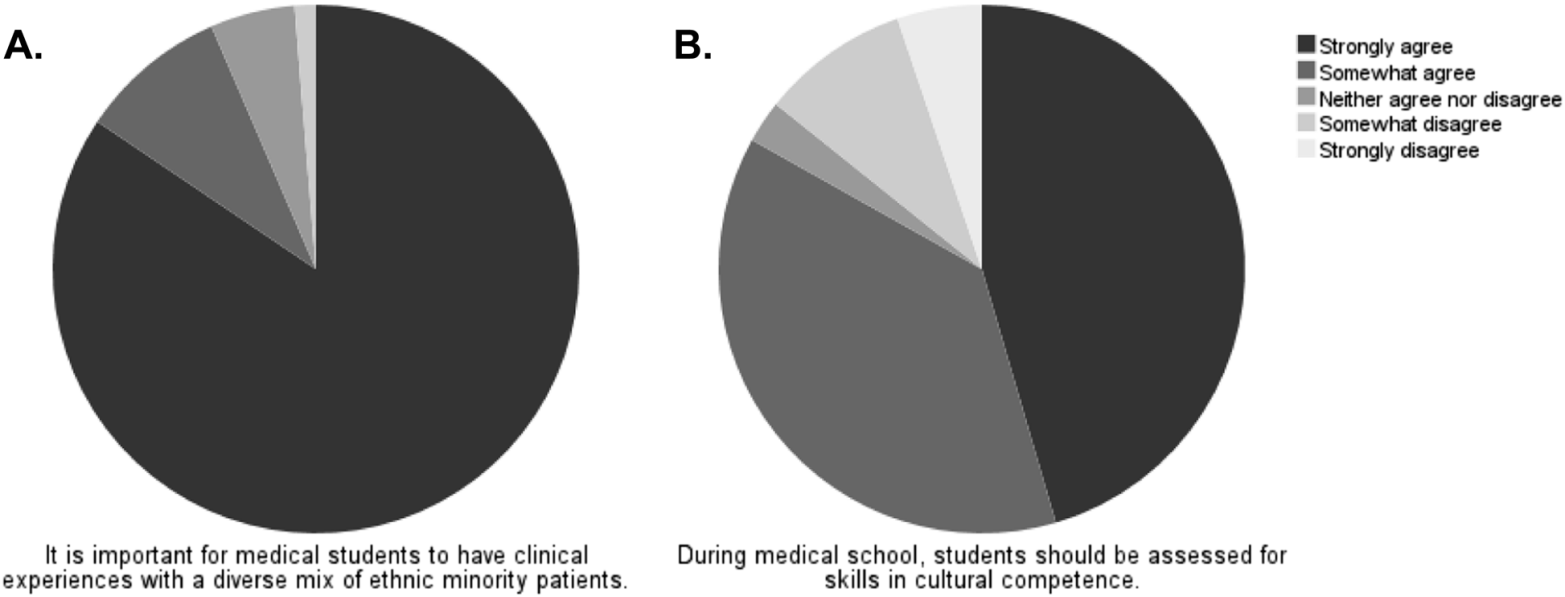
Participant attitudes to cross-cultural care were assessed using a 5-point Likert scale from strongly disagree to strongly agree. **A** Participants were asked whether they felt it is important for medical students to have clinical experiences with a diverse mix of ethnic minority patients. “Strongly disagree” (0%), “somewhat disagree” (1.3%), “neither agree nor disagree” (5.2%), “somewhat agree” (9.1%), “strongly agree” (84.4%). **B** Participants were asked whether during medical school, students should be assessed for skills in cultural competence. “Strongly disagree” (5.2%), “somewhat disagree” (9.1%), “neither agree nor disagree” (2.6%), “somewhat agree” (37.75%), “strongly agree” (45.5%). Total number participants, (*N* = 77)

Participant attitudes towards cross-cultural care were assessed. The majority of participants agreed that it is important to have clinical experience with diverse patient populations (Fig. 4A) (93.5%). Furthermore, 83.2% of participants agreed that students should be assessed for their skills in cultural competence (Fig. 4B).

Finally, participants were invited to share ideas of how cross-cultural care may be further incorporated into their learning. Fourteen respondents put forward their thoughts on ways to deliver effective cross-cultural care within their curriculum. These may be considered under three broader categories—the method of delivery, the resources used, and the content delivered. Respondents felt methods of delivery should include specified lectures and/or modules on cross-cultural care, clinical sessions with patients from diverse backgrounds, and opportunities for involvement in community initiatives delivering care to minority populations. Respondents highlighted the need for learning resources that are inclusive of diverse patient populations. Finally, respondents highlighted a need for specific training in key content areas, including unconscious bias and working with medical interpreters.

## Discussion

This is one of the first studies in a European context to evaluate medical students’ preparedness to care for diverse patient populations using a validated survey tool and identifies areas of need to equip students to provide high-quality cross-cultural care. Previous studies have provided limited insight into the medical student perspective on aspects of cross-cultural training, usually as part of wider studies focusing on the perceptions of clinicians or schools delivering training [9, 12].

The self-reported preparedness points to a specific lack of experience engaging with patients from diverse backgrounds. With a majority of respondents reporting proficiency in cross-cultural skills assessed in this survey, this may reflect an under-confidence in self-reported preparedness of Irish medical students. The difference in preparedness between self-identified ethnic groups, whereby ethnic minority students reported greater preparedness in cross-cultural care, may be due to a shared experience of being minoritised in society and finding a commonality between being from cultures outside of the ethnic majority. Other studies have found similar results, with participants that identify as ethnic minority or even sexual minority reporting greater preparedness to care for patients from different cultural backgrounds and different sexual orientations [8, 18]. It may suggest that these experiences may be collected, shared, and taught in cross-cultural education, so that future clinicians are able to better understand their diverse patients and ultimately deliver better care.

Preparedness to care for LGBTQIA + patients and patients with disabilities were included in the survey, as these groups also often face barriers to care. While few survey respondents felt prepared to care for these communities, the figures were similar to those collected in the USA and Taiwan [6, 7]. This suggests an area to improve upon when cultivating student preparedness to care for other diverse populations, populations that are often neglected or discriminated against in the health care setting despite a potentially shared culture or ethnicity [18].

The Skills section highlighted specific areas of student concern, which can direct the development of future medical curricula on cross-cultural training. Participants in this study identified areas where they felt least skilled in delivering cross-cultural care, including identifying religious or cultural beliefs affecting clinical care and working effectively with a medical interpreter. They highlighted experiences outside the formal medical curriculum as most preparatory in building their cross-cultural competence. Research indicates that the informal or “hidden” curriculum, where students encounter a variety of patient populations and learn through direct observation, immersion, and interaction with these diverse groups, plays a crucial role in developing their cultural competence. This unintentional learning process is essential in enhancing their ability to effectively work across different cultural contexts [19]. This suggests that schools should offer and encourage elective opportunities or volunteering placements within diverse communities, as they are a rich source of cross-cultural education. Unfortunately, medical schools in Ireland are often limited by geographical location and availability of clinical placements. However, this may be an area schools can improve upon as the Irish population continues to diversify rapidly.

Despite varying cross-cultural training programs implemented in Irish medical schools, many participants in this survey felt they had not received training in cross-cultural care during their time in medical school, nor was it felt to be a priority in their curriculum. Further reinforcement of these programmes should be implemented across all years of medical school, via both theoretical and practical means. As per participant suggestions put forth in this survey, lectures, small group sessions, involvement in local community programmes, and dedicated cross-cultural clinical sessions could be implemented to enhance cultural competency. A cultural humility approach has been shown to be beneficial, which incorporates self-reflection in cross-cultural training [12]. A scoping review by Brottman et al*.* [19], revealed eleven cross-cultural educational methods to cultivate cultural competence, whilst Liu et al*.* [20], demonstrated the ways in which the hidden curriculum can influence cross-cultural competence. From these studies, multiple methods of cultivating cross-cultural competence can be utilised, and there is no method has been proven superior to another [19].

The majority of participants agreed that they should be assessed for skills in cultural competence during medical school training. Schools may look to assess this in Objective Structured Clinical Exam (OSCE) stations. There have been previous calls for greater use of objective measures of assessment of cultural competence in the literature to date [6, 8, 11]. A recent review by Deliz. et al. [21] found that the most commonly adopted assessment modality of cross-cultural care training in medical schools were pre- and post-training self-assessment surveys, but other forms of assessment included objective measures, namely knowledge-based tests and standardised patient encounters. It is unknown whether the medical schools listed in our study have implemented objective assessments for cultural competence among their student population.

The Attitudes section suggests that survey participants have encountered negative or dismissive attitudes towards cross-cultural care in clinical settings. This follows findings from UK studies, which revealed that ethnic minority students specifically felt isolated and subject to stereotyping by clinicians whilst on placement [12, 22]. This suggests that clinical staff should also be exposed to cross-cultural training as role models for future health care professionals [19]. Fortunately, positive attitudes greatly outweighed negative attitudes amongst the participants’ own peer groups.

One limitation of this study was the low number of responses received, which may have been impacted by our method of recruitment and timing of our data collection. Our data were collected through indirect social media channels, therefore the number of medical students that had the potential to interact with our survey was unknown. There was a low response rate from some institutions compared to others, namely TCD, RCSI, and UCC, again likely due to method of recruitment, thus the data cannot be taken to represent all undergraduate and graduate medicine courses in the ROI. The data collection took place in the latter half of the final year, a time when student anxiety regarding final exams is high. This may have impacted the rate of participation observed in our study. While there is no public data available regarding the ethnic makeup of the medical student population in Ireland, our survey received responses representing self-identified majority and self-identified minority student perspectives. Though this may not reflect the national average, this response ratio ensured representation from both cohorts. The interpretation of data inferred from ethnicity differences cannot be overstated due to the low total number of responses. Also, it should be noted that this study asked students to self-identify as ethnic minority or majority, which may inherently pose difficulty for some.

Students engaging in a survey on cross-cultural care are likely interested in this area of medical education, which may influence the responses. This survey asked students to self-report their feelings of preparedness and skill and may not be a true reflection of their abilities. Students may feel unprepared at this stage of their career due to “imposter syndrome” or anxiety about entering the workforce, which may create a negative self-perception bias [23]. There is limited data published regarding how the schools represented in this survey implement their training in cross-cultural care. Finally, this survey tool relies on participant recall, introducing potential for recall bias as observed in similar studies [7]. Suggestions for further research include repeating this survey with alternative recruitment methods to boost response rates and collect data representative of all ROI medical institutions, assessing students’ preparedness for diverse patient populations during different stages of their medical education. The preparedness of medical students to care for patients with disabilities and/or patients from LGBTQIA + communities should be further explored. Finally, it would be advisable to assess non-hospital consultant doctors’ (NCHDs) preparedness to care for diverse patient populations in Ireland.

## Conclusions

This was the first study assessing the perceptions of final-year medical students across Irish universities in their preparedness, skill, and attitudes towards cross-cultural care. This survey has helped to clarify the student perspective on current cross-cultural training employed by medical schools, with students reporting an unpreparedness to care for diverse patient cohorts. It highlights areas in which students do not feel adequately trained to deliver cross-cultural care. Most students have a positive perception of cross-cultural competence and feel it is important to incorporate cross-cultural competence into their education to ensure the delivery of equitable health care to diverse patient cohorts. Students expressed how they hoped to see more cross-cultural competency training, including further lectures, modules, and clinical sessions added to their curriculum. This survey has highlighted areas of medical education that students desire further training in to develop their skills in cross-cultural competence.

### Supplementary Information

**Supplementary Material 1.**

## Data Availability

The datasets generated and analysed during the current study are not publicly available due to the potential for individual privacy to be compromised but are available from the corresponding authors on reasonable request.

## Abbreviations

CCCS: Cross-Cultural Care Survey
HSE: Health Service Executive
LGBTQIA +: Lesbian, Gay, Bisexual, Transgender, Queer, Intersex, Asexual +
NCHD: Non-Consultant Hospital Doctor
NUIG: National University of Ireland, Galway
OSCE: Observed Structured Clinical Exam
RCSI: Royal College of Surgeons in Ireland
ROI: Republic of Ireland
TCD: Trinity College Dublin
UCC: University College Cork
UCD: University College Dublin
UK: United Kingdom
UL: University of Limerick
USA: United States of America
