# Supplementary material for: Perceptions of and preparedness for cross-cultural care: a survey of final-year medical students in Ireland
Source: BMC Med Educ. 2024 Apr 29;24:472. doi: 10.1186/s12909-024-05392-4 (PMC11059656; doi:10.1186/s12909-024-05392-4)
Supplement: Supplementary file 1 — Supplementary Material 1. [file 12909_2024_5392_MOESM1_ESM.docx]

Supplementary file 1 (.pdf)

Transcript of survey used in research study. This includes the definition used for “ethnic minority” in this research study. The following domains were included in this survey: demographics, preparedness, skill training & education, and attitudes.

# Definition

**Please read the following carefully before proceeding:**

According to the most recent Irish census data in 2016, 91.7% of the population in Ireland identifies as White.

For the purposes of this study, an ethnic minority is defined by **any one (or more)** of the following:

i. Non-White

ii. Non-English speaker

iii. Irish Traveller/Roma

# Demographics

1. Are you a final year medical student in the Republic of Ireland?

Yes

No

1. Please indicate your medical school:

NUIG

RCSI

TCD

UCC

UCD

UL

Prefer not to say

1. Please indicate the gender you identify as:

Male

Female

Non-binary

Prefer not to say

# Preparedness

1. Please indicate **how prepared you feel to care for patients** in the following:
2. To care for patients **in general**?
3. To care for patients **who are members of racial and ethnic minorities**?
4. To care for patients **who are members of the Irish Traveller community**?
5. To care for patients **who have limited proficiency in the English language**?
6. To care for patients **who are new immigrants**?
7. To care for patients **whose religious beliefs affect treatment**?
8. To care for patients **who are LGBTQIA+**?
9. To care for patients **who are persons with disabilities**?

| 1 | 2 | 3 | 4 | 5 |
| --- | --- | --- | --- | --- |
| Very unprepared | Somewhat unprepared | Neither prepared nor unprepared | Somewhat prepared | Very prepared |

# Skill

1. Please rate **how skilled you are at each of the following:**
2. Identifying how well a patient can read or write English
3. Identifying how well a patient understands the English that is being spoken to them
4. Identifying religious beliefs and cultural customs that might affect clinical care
5. Adapting my communication style to accommodate a patient;s needs
6. Building rapport with patients from an ethnic background different to my own
7. Working effectively with a medical interpreter

| 1 | 2 | 3 | 4 | 5 |
| --- | --- | --- | --- | --- |
| Very unskilled | Somewhat unskilled | Neither skilled nor unskilled | Somewhat skilled | Very skilled |

# Training and Education

1. **The following experiences were useful in preparing** **me to care for ethnic minority patients**:
2. Preclinical years
3. Clinical years
4. Electives
5. Experiences prior to, or outside of, the formal medical curriculum

| 1 | 2 | 3 | 4 | 5 |
| --- | --- | --- | --- | --- |
| Strongly disagree | Somewhat disagree | Neither agree nor disagree | Somewhat agree | Strongly agree |

1. **Please describe the experience(s) during medical school that have been most useful in preparing you to care for ethnic minority patients?** (optional)

For the following questions, please note **cross-cultural training** refers to any formal or informal training in delivering cross cultural care.

**Cross-cultural care** is the provision of care to patients from diverse ethnic and/or cultural backgrounds.

1. **"During my medical school training, I have been exposed to the following: "**
2. Practical experience caring for diverse patient populations
3. Cross-cultural training during medical school
4. Good role models or mentors for cross-cultural care among faculty
5. Positive attitudes about cross-cultural care among clinicians on placement
6. Negative/dismissive attitudes about cross-cultural care among clinicians on placement
7. Positive attitudes about cross-cultural care among your fellow students
8. Negative/dismissive attitudes about cross-cultural care among your fellow students

| 1 | 2 | 3 | 4 | 5 |
| --- | --- | --- | --- | --- |
| Strongly disagree | Somewhat disagree | Neither agree nor disagree | Somewhat agree | Strongly agree |

1. **For the following questions, please indicate your response:**
2. My medical school has incorporated cross-cultural issues into teaching and clinical care.
3. My medical school makes learning about the care of ethnic minority patients a priority for medical education.

| 1 | 2 | 3 | 4 | 5 |
| --- | --- | --- | --- | --- |
| Strongly disagree | Somewhat disagree | Neither agree nor disagree | Somewhat agree | Strongly agree |

1. **Please describe what types of formal educational or other training opportunities your medical school might consider to help prepare you to care for diverse patient populations.** (Optional)

# Attitudes

1. **For the following questions, please indicate your response:**
2. Across the healthcare system, ethnic minority patients generally receive lower quality care because of their race/ethnicity.
3. Unconscious racial stereotyping by doctors is a problem.
4. Unconscious stereotyping by doctors is a problem for the Irish Traveller community.
5. Clinicians acquire cultural biases during medical training.
6. It is important to reflect on how our own biases, conscious or unconscious, may affect patient care.
7. Learning about the health beliefs of patients from different cultures is an important part of being a good doctor.
8. Understanding a patient’s social circumstances is vital to developing a treatment plan.
9. It is important for medical students to have clinical experiences with a diverse mix of ethnic minority patients.
10. During medical school, students should be assessed for skills in cultural competence.

| 1 | 2 | 3 | 4 | 5 |
| --- | --- | --- | --- | --- |
| Strongly disagree | Somewhat disagree | Neither agree nor disagree | Somewhat agree | Strongly agree |
